# Imaging features of CT and MR in hepatic perivascular epithelioid cell tumor: case series and literature review

**DOI:** 10.3389/fonc.2025.1616755

**Published:** 2025-08-11

**Authors:** Ning Li, Hui Liu, Jing Chen, Yu Kang, Jing Zhou, Janhua Wang

**Affiliations:** ^1^ Department of Radiology, Affiliated Hospital of Nanjing University of Chinese Medicine, Nan Jing, China; ^2^ Department of Ultrasonic Medicine, Affiliated Hospital of Nanjing University of Chinese Medicine, Nan Jing, China

**Keywords:** liver, PEComas, CT, MR, hepatic

## Abstract

**Objective:**

To retrospectively analyze the clinical and imaging data of 4 patients with hepatic PEComa of different pathological grades, and to review relevant literature, aiming to enhance clinical awareness of this disease and provide support for preoperative diagnosis.

**Methods:**

A retrospective analysis was conducted on 4 cases of patients with hepatic PEComa who were confirmed by surgery and biopsy in our hospital. All patients had complete clinical data, including laboratory tests, abdominal imaging studies, as well as pathological and immunohistochemical examinations.

**Results:**

Four cases of hepatic PEComa were identified. All patients were female, aged between 51 and 62 years. The largest diameter of the lesions ranged from 2.4 cm to 20.0 cm. All four lesions presented a round or oval shape with well-defined margins. Three lesions showed homogeneous density/signal, while one lesion displayed heterogeneous density with extensive necrosis. All four cases had a rich blood supply, characterized by significant enhancement in the arterial phase. In two cases, curved and dilated feeding arteries were observed at the periphery of the lesions, and in one case, early visualization of the hepatic vein occurred during the arterial phase. Immunohistochemical staining was positive for HMB45 (+/++), Melan-A (+/++), and SMA (+/++) in all four cases.

**Conclusion:**

Hepatic PEComa is a rare tumor with no typical clinical manifestations, and laboratory tests usually show no significant abnormalities. CT and MRI are valuable in preoperative diagnosis. When a lesion exhibits prominent arterial phase enhancement along with thickened and tortuous vessels, and there are no specific findings in the clinical history and laboratory tests, hepatic PEComa should be included in the differential diagnosis.

## Introduction

Perivascular epithelioid cells (PECs) are a unique cell population located around blood vessels, usually arranged in a radial pattern. According to the World Health Organization (WHO), perivascular epithelioid cell tumors (PEComas) are defined as a separate category of mesenchymal neoplasms. These tumors are characterized by the presence of perivascular epithelioid cells, which can be identified through their histological features and immunophenotypic profiles (1). Hepatic PEComa is an extremely rare condition (2). Currently, there are no well - established specific clinical manifestations or imaging diagnostic criteria for hepatic PEComa. As a result, it is often misdiagnosed as other liver diseases, such as hepatocellular carcinoma(HCC), hepatocellular adenoma(HCA), or focal nodular hyperplasia(FNH) (3). To address this issue, this study retrospectively analyzed the clinical and imaging data of 4 patients with hepatic PEComa of different pathological grades and reviewed relevant literature. The aim was to improve clinical awareness of this disease and aid in preoperative diagnosis.

## Materials and methods

This retrospective study adhered to the ethical guidelines of the 1975 Declaration of Helsinki and was approved by the ethics committee of our hospital. Given the retrospective nature of the research, the requirement for informed consent was waived.

### Patients

A retrospective analysis was performed on 4 patients with hepatic PEComa who were confirmed by surgical resection and biopsy at our hospital between November 2017 and August 2024. The inclusion criteria were as follows: all patients had complete clinical data, including blood biochemical analyses, tumor marker assessments, hepatitis virology evaluations, abdominal imaging studies, as well as pathological and immunohistochemical examinations. The exclusion criterion was incomplete imaging and clinical data.

### Imaging and pathological examination

The CT scan was performed using a Philips Brilliance 64 iCT scanner. The scanning parameters were set as follows: tube voltage at 120 kV, tube current ranging from 200 to 400 mA, slice thickness and spacing of 1.0 mm, pitch between 0.984 and 1.375, and a rotation speed of 0.5 seconds per rotation. For the enhanced CT scan, a nonionic contrast agent, iodine hexylide (350 mg I/ml), was administered at a volume of 100–120 ml. The arterial phase was acquired approximately 35 seconds after injection, the portal phase around 70 seconds, and the delayed phase about 120 seconds. The scanning range covered from the apex of the diaphragm to the inferior border of the pubic symphysis.

The MR examination utilized a Siemens 3.0T MRI scanner with an 8-channel abdominal coil. The patient was placed in a supine position, and the scanning range extended from the diaphragm to the inferior margin of the liver. The contrast agents used were Gd-EOB-DTPA or Gd-DTPA, injected into the antecubital vein of the arm at a flow rate of 1.0 ml/s, with a dose of 0.025 mmol/kg. Subsequently, an equal volume of saline solution was injected at the same flow rate to flush the line. For both non-contrast and dynamic contrast-enhanced imaging, axial liver volume acceleration scans were conducted with a repetition time (TR) of 2.8 ms, an echo time (TE) of 1.2 - 1.3 ms, a flip angle of 20°, a field of view (FOV) of 380.0 mm × 380.0 mm, a slice thickness of 5 mm, and a slice gap of 1 mm. The contrast agent was administered at 20 seconds, 55 seconds, 2–5 minutes, and 20 minutes to define the arterial phase, portal vein phase, transitional phase, biliary phase, and hepatic phase, respectively.

All surgical specimens and biopsy tissues obtained from the patients underwent routine pathological examination and immunohistochemical staining.

### Imaging analysis

Two experienced radiologists, who were blinded to the pathological results, jointly evaluated all imaging data. In case of any discrepancies, a consensus was reached through joint discussion. The evaluation included the lesion’s location, morphology, size, density/signal characteristics, enhancement pattern, and its relationship with adjacent vascular structures.

## Results

### Patients

Four cases of hepatic PEComa were identified, all of whom were female. Their ages ranged from 51 to 62 years, with a median age of 55 years. One case was incidentally detected during a routine physical examination. Another was discovered during an abdominal CT scan for vulvar cancer. One patient presented with right upper abdominal discomfort, and the last patient reported fatigue and weight loss. The serology results for hepatitis viruses were negative in all cases. Tumor markers showed mild elevations in two cases (CA125 levels were 38 and 35.6, respectively; normal range < 35), and no other abnormalities were found.

### Pathological data

Two cases were diagnosed as benign hepatic PEComa. One case was classified as hepatic PEComa with malignant potential due to the presence of significantly atypical tumor cells and individual nuclear division figures. Additionally, one case was determined to be malignant hepatic PEComa. Immunohistochemical staining was positive for HMB45 (+/++), Melan-A (+/++), and SMA (+/++) in all four cases. Notably, the Ki67 proliferation index of the malignant liver PEComa exceeded 70%, which was significantly higher than that of the other three cases (**Table 1**).

**Table 1:** 4 cases of hepatic PEComa pathology and immunohistochemistry.

Case	Pathology	Immunohistochemistry				
		HMB45/Melan-A/SMA	CD34	S-100	Arginase-1	Ki67
1	Hepatic PEComa	++/+/++	+	–	+	5%
2	Hepatic PEComa	++/++/+	+	+	–	5%
3	Hepatic PEComa with malignant potential	+/+/+	+	–	–	2%
4	Malignant hepatic PEComa	+/+/+	–	–	–	70%

PEComa, perivascular epithelioid cell tumors; HMB-45, human melanoma black-45; SMA, smooth muscle actin.

### Imaging data

**Figure 1:**
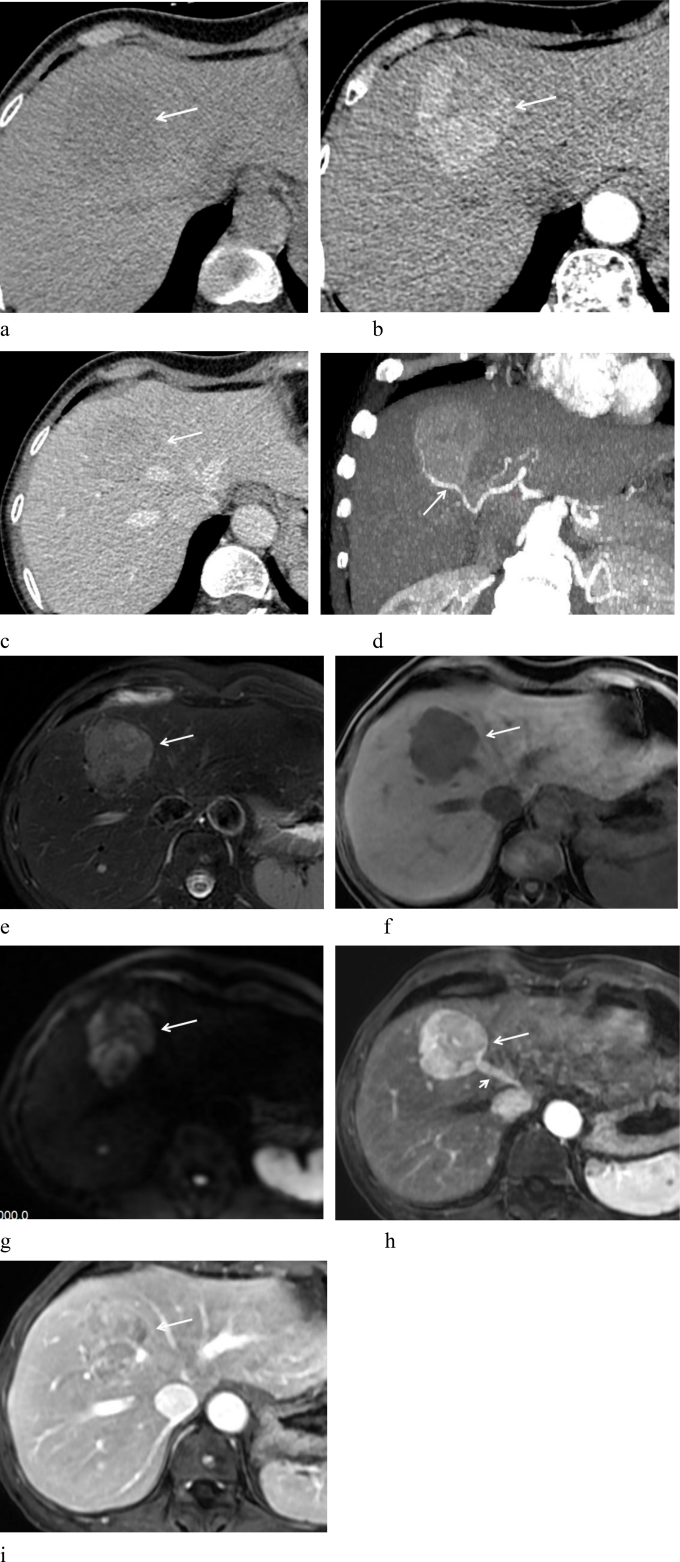
A 62-year-old woman with hepatic PEComa of malignant potential. **(a)** Non-contrast CT scan shows homogeneous mild hypodensity. **(b)** Arterial phase demonstrates marked heterogeneous enhancement. **(c)** Portal phase exhibits rapid contrast washout. **(d)** Arterial phase MIP image depicts a feeding artery (arrow) surrounding the lesion. **(e)** T2-weighted imaging (T2WI) reveals high signal intensity with a low-signal “pseudo-capsule” at the margin and well-defined borders. **(f)** T1-weighted imaging (T1WI) shows low signal intensity. **(g)** Diffusion-weighted imaging (DWI) displays high signal intensity. **(h)** Arterial phase shows prominent enhancement, with early opacification of the draining hepatic vein (short arrow), suggesting an intra-lesional arteriovenous fistula. **(i)** Portal phase shows rapid washout, accompanied by peripheral “pseudo-capsule” enhancement (arrow).

**Figure 2:**
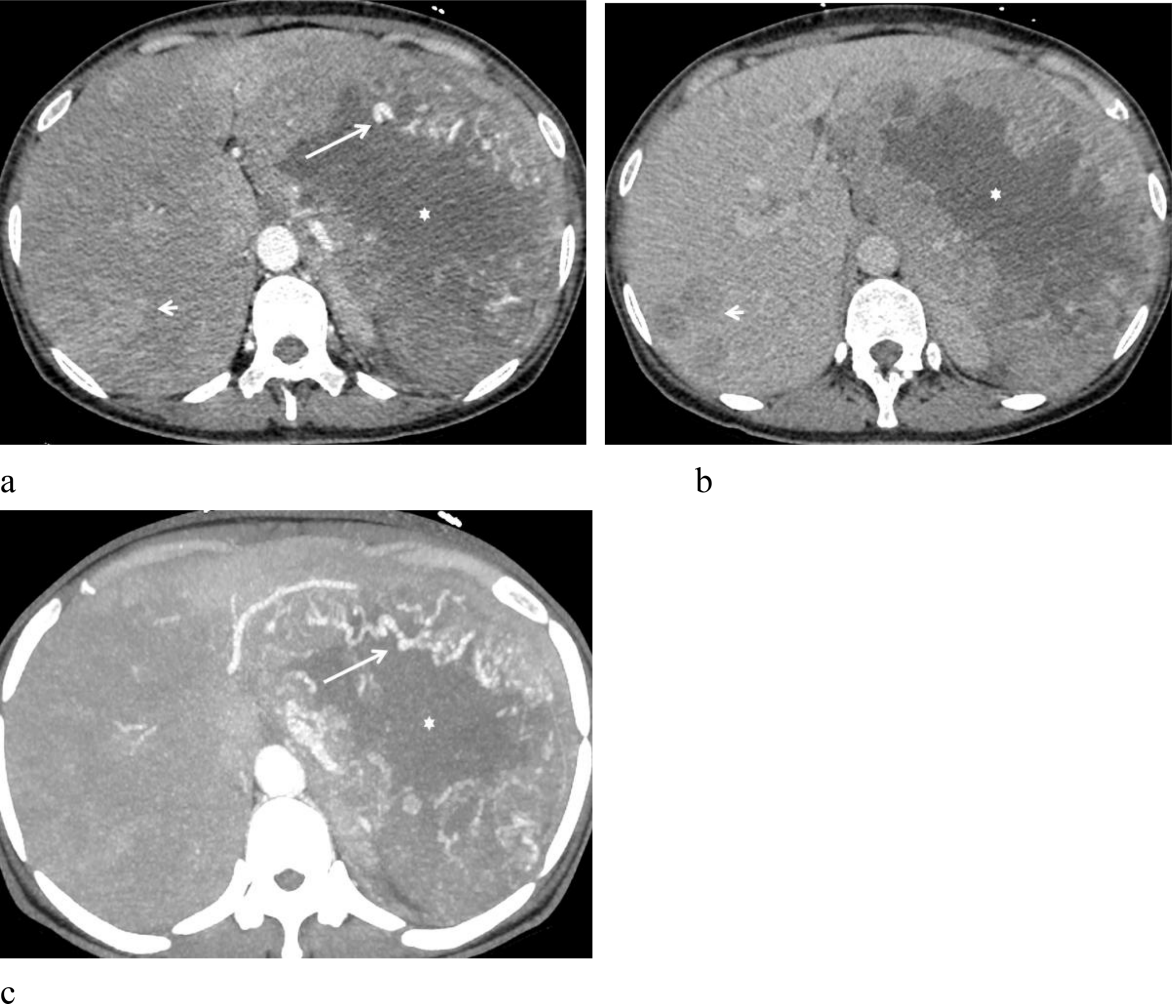
A 51-year-old woman with malignant hepatic PEComa. **(a)** Arterial phase imaging reveals a large left hepatic lobe lesion surrounded by multiple tortuous and dilated feeding arteries (arrow). The lesion shows marked heterogeneous enhancement, with a substantial low-density necrotic area (star). Several similar hyperenhancing lesions are also observed within the liver (short arrow). **(b)** Portal phase demonstrates rapid contrast washout. **(c)** Arterial phase MIP image highlights multiple tortuous, dilated feeding arteries (arrows) encircling the lesion.

One patient underwent contrast-enhanced MR imaging, two patients underwent both contrast-enhanced CT and MR imaging, and one patient only had contrast-enhanced CT (**Table 2**). Among them, three patients had solitary lesions, and one had multiple lesions. The largest diameter of the lesions ranged from 2.4 cm to 20.0 cm. The lesions were located in liver segment VI in two cases. In one case, the lesion involved both liver segments IV and VIII. Another patient had multiple lesions distributed throughout the liver, with larger masses mainly located in the left lobe. All four lesions were round or oval in shape with well-defined margins. Three lesions showed homogeneous density/signal, while one lesion had heterogeneous density with extensive necrosis. After contrast enhancement, all four cases showed a rich blood supply, characterized by prominent arterial phase enhancement. In two cases, curved and dilated feeding arteries were observed at the periphery of the lesions. Two cases showed high arterial phase enhancement with rapid washout in the portal phase, while the other two cases showed high arterial phase enhancement with slow washout. One case exhibited early visualization of the hepatic vein during the arterial phase, suggesting the possible formation of a hepatic artery-vein fistula within the tumor. One case presented with peripheral “pseudo-capsule” enhancement (**Figure 1**), and one case showed low signal intensity during the hepatobiliary phase. Initially, three cases were misdiagnosed as HCC, and one case was misdiagnosed as a metastatic tumor. Notably, case 4 was diagnosed as malignant hepatic PEComa (**Figure 2**), during the three-month postoperative follow-up, the number and size of the liver lesions in this patient increased, which confirmed ntrahepatic metastasis of PEComa.

**Table 2:** 4 cases of hepatic PEComa imaging findings.

Case	Age (year)	Exam	Location	Long axis (cm)	MR scan	CT scan	CT/MRI Enhancement pattern	Preoperative diagnosis
1	55	MR	Segment VI	2.4	T1WI hypointense; T2WI and DWI hyperintense; well - defined margins, homogeneous signal	–	Arterial phase: intense enhancement; Portal and delayed phases: slow washout	HCC
2	55	CT/MR	Segment VI	3.1	T1WI hypointense; T2WI and DWI hyperintense; well - defined margins, homogeneous signal	Hypodense lesion with well - defined margins and homogeneous density	Arterial phase: intense enhancement; Portal and delayed phases: slow washout; Hepatobiliary phase: hypointense	Metastatic tumor
3	62	CT/MR	Across segments IV and VIII	5.1	T1WI hypointense; T2WI and DWI hyperintense; well - defined margins, homogeneous signal	Hypodense lesion with well - defined margins and homogeneous density	Arterial phase: intense enhancement; Portal phase: rapid washout; Surrounded by curved and dilated feeding arteries; Early visualization of the middle hepatic vein in the arterial phase, suggesting possible hepatic arteriovenous fistula	HCC
4	51	CT	Multiple	20.0	–	–	Arterial phase: intense enhancement; Portal phase: rapid washout; Surrounded by curved and dilated feeding arteries	HCC with multiple intrahepatic metastases.

CT, computerized tomography; MRI, magnetic resonance imaging; T1WI, T1-weighted imaging; T2WI, T2-weighted imaging; DWI, diffusion weighted imaging; HCC, hepatocellular carcinoma.

## Discussion

PEComas are rare mesenchymal tumors, first described by Bonetti et al. in 1992 (4). They are characterized by a unique composition of vascular perivascular epithelioid cells with diverse morphological and immunohistochemical features. The PEComa family encompasses angiomyolipoma (AML), clear cell sugar tumor of the lung (CCST), lymphangioleiomyoma/lymphangioleiomyomatosis (LAM), and a group of immunohistochemically similar tumors, including primary extrapulmonary sugar tumor, clear-cell myomelanocytic tumor (CCMMT) of the falciform ligament/ligamentum teres, abdominopelvic sarcoma of PECs, and PEComa arising in various soft tissue and visceral sites (5, 6). PEComas can occur in many anatomical locations, but they predominantly arise in the uterus, retroperitoneum, abdominopelvic region, and gastrointestinal tract. Hepatic PEComas, in particular, are extremely rare (7).

Hepatic PEComa predominantly affects middle-aged women (8) and usually presents without distinctive clinical symptoms or signs, often being incidentally detected during routine physical examinations (9). Larger tumors may cause abdominal pain due to compression of adjacent tissues or result in rupture and hemorrhage. Unlike HCC, this condition is not associated with hepatitis or liver cirrhosis, and tumor markers are typically negative (8, 10). The cases in our study align well with these characteristics. All patients were female, with a median age of 55 years. One case was detected during a routine physical examination, another during an abdominal CT scan for vulvar cancer, one patient presented with right upper abdominal discomfort, and the last reported fatigue and weight loss. Hepatitis virus markers were negative in all cases; while two cases showed mild elevations in CA125 levels, other tumor markers remained within normal ranges.

Histologically, hepatic PEComa is mainly composed of proliferating epithelioid and spindle cells. The tumor cells are polygonal, with translucent cytoplasm containing eosinophilic particles, and thick-walled blood vessels are observable within the tumors. Epithelioid cells are radially arranged around these thick-walled blood vessels, and feather-like collagen fibers can be identified (11). Immunohistochemically, PEComa tumor cells exhibit features of perivascular epithelioid cell differentiation, similar to melanocytes, neuroendocrine cells, and smooth muscle cells. They commonly test positive for HMB-45, Melan-A, and SMA (11–13). Tumors with a predominantly epithelial composition tend to express melanocyte markers, whereas those with a spindle-shaped morphology often show myogenic cell markers (SMA is common, Desmin is less so) and lack hepatic markers such as Hep and LFABP. Among these, HMB-45 is the most sensitive diagnostic marker for PEComa. In our study, all four cases were positive for HMB-45 (+/++), Melan-A (+), and SMA (+/++), consistent with previous reports. Notably, the malignant liver PEComa case showed a Ki67 expression level exceeding 70%, significantly higher than the other three cases. Ki67, a protein involved in cell proliferation and expressed in all active cell cycle phases except G0, is located exclusively in the nuclei of proliferating cells. Its expression level is closely correlated with tumor grade and biological behavior (14). The Ki67 expression rate serves as a key indicator of early tumor cell proliferation, and as tumor cells continue to divide, increased Ki67 expression reflects higher pathological grades and malignancy (15).

The imaging characteristics of hepatic PEComa vary depending on the heterogeneity of tissue components (10), and neither CT nor MRI shows high specificity for diagnosis (3, 16, 17). On CT, it typically appears as a low or mildly hypodense mass; on MRI, it shows a slightly hypointense signal on T1-weighted images (T1WI), a mildly hyperintense signal on T2-weighted images (T2WI), and high signal intensity on diffusion-weighted imaging (DWI), usually with well-defined margins (18). The presence of fat within a hepatic lesion is a notable feature, strongly suggesting a diagnosis of hepatic PEComa (19). MRI is more sensitive than CT in detecting fat components, especially with the aid of fat suppression techniques (20). However, the fat content in tumors can range from 10% to 90% (21), and for tumors with low fat content, both CT and MRI may fail to clearly demonstrate this feature, posing diagnostic challenges. In our four cases, all had minimal fat content, and no obvious fat components were detected on CT or MRI. Three cases showed homogeneous density/signal, while the malignant case exhibited heterogeneous density with extensive areas of low-density necrosis.

The CT and MR enhancement patterns of hepatic PEComa share common features, characterized by intense arterial phase enhancement and thickened vessels within and around the lesion (16, 17), sometimes accompanied by peripheral draining veins (21). Gao X et al. (22) reported two enhancement patterns, “fast in and fast out” and “fast in and slow out”, in 11 cases of hepatic PEComa. Nie P et al. (17) described four enhancement patterns in 22 cases: arterial phase enhancement with rapid washout (n= 9), arterial phase enhancement with slow washout (n= 7), arterial phase enhancement with persistent late-phase enhancement (n= 4), and unspecified heterogeneous enhancement (n= 2). Literature also indicates that hepatic PEComa shows low signal intensity during the hepatobiliary phase, which corresponds to its pathological features (23). In our study, all four hepatic PEComa cases had abundant vascularity and prominent arterial phase enhancement. Two cases showed vascularity both at the periphery and within the tumor. One case showed early visualization of the middle hepatic vein during the arterial phase, suggesting the presence of a hepatic arteriovenous fistula. Two cases showed high arterial phase enhancement with rapid portal phase washout, and one of these lesions exhibited a “pseudo-capsule” effect. Two cases had high arterial phase enhancement with slow washout, and one case showed low signal intensity during the hepatobiliary phase.

Although most hepatic PEComas are benign, 4% to 10% of cases are malignant (24). Folpe et al. (25) first proposed diagnostic criteria for potentially malignant PEComas, including tumor size >5cm, infiltration of surrounding tissues, vascular invasion, high nuclear grade, high cellularity, necrosis, and a mitotic figure >1/50 high power field. Based on these criteria, PEComas are classified into three categories: (1) malignant (meeting two or more of the above criteria); (2) benign (meeting none of the criteria); and (3) with malignant potential (characterized by a tumor maximum diameter > 5 cm or nuclear hyperplasia) (26). In our study, cases 1 and 2 were classified as benign. Case 3, with the largest lesion diameter of 5.2 cm, significant atypia in several tumor cells, and individual nuclear divisions, was diagnosed as PEComa with malignant potential, after three months of interventional treatment, follow-up showed no significant tumor growth. Case 4 was diagnosed as malignant hepatic PEComa, which met the above malignant diagnostic criteria, during the three-month postoperative follow-up, the number and size of the liver lesions in this patient increased, which confirmed ntrahepatic metastasis of PEComa.

The differential diagnosis of hepatic PEComa is relatively straightforward when fat tissue is present in the lesion, increasing the likelihood of diagnosing it as either hepatic PEComa or angiomyolipoma (AML) (21, 27). However, the absence of fat makes preoperative diagnosis difficult, often leading to misdiagnosis as HCC, HCA, or FNH. Misdiagnosis as primary HCC is the most common (28). In our cohort, three cases were misdiagnosed as HCC, and one as a metastatic tumor. HCC predominantly affects males, is often associated with a history of hepatitis B or C and liver cirrhosis, and typically shows elevated AFP levels. It exhibits intense arterial phase enhancement followed by rapid washout in the portal and delayed phases, and hepatic portal vein thrombus formation is common. HCA mainly occurs in young women, often related to the use of contraceptive pills or steroid medications, and frequently presents with intra-tumoral hemorrhage and necrosis. FNH more common in middle-aged and younger women, lacks a capsule, shows rapid inflow enhancement with gradual outflow enhancement, may have central star-shaped scars with delayed enhancement, and lesions usually show equal or slightly increased signal intensity in the hepatobiliary phase.

Surgical resection is the preferred and effective treatment for primary hepatic PEComa (29). However, due to the limited number of retrospective case studies, a standardized treatment protocol has not been established. Sanfilippo R et al. (30) suggested that mTOR inhibitors may be effective for advanced or metastatic PEComa patients, while Kirste S et al. (31) proposed stereotactic body radiation therapy as a potential treatment option. Guan H et al. (32) also reported promising clinical outcomes with interventional therapy. In our study, two cases underwent surgical resection, one case was successfully treated with interventional therapy, and one case was diagnosed by biopsy without subsequent specific treatment.

Our study has several limitations. First, as a retrospective study, it was subject to selection and reporting biases. Second, the sample size was small, with only four cases from a single center. Future multi-center studies with larger sample sizes are needed to further validate our findings.

In conclusion, hepatic PEComa is a rare tumor that predominantly affects middle-aged women and lacks typical clinical manifestations. Laboratory tests usually do not reveal specific abnormalities. Although CT and MRI play a role in preoperative diagnosis, due to the tumor’s multi-directional differentiation, misdiagnosis rates remain high. When a lesion shows marked arterial phase enhancement with thickened and tortuous vessels, and there are no specific findings in the clinical history and laboratory tests, hepatic PEComa should be considered. A definitive diagnosis ultimately relies on pathological and immunohistochemical examinations.

## Data Availability

The original contributions presented in the study are included in the article/supplementary material. Further inquiries can be directed to the corresponding authors.
